# *In vitro* Induction and Phenotypic Variations of Autotetraploid Garlic (*Allium sativum* L.) With Dwarfism

**DOI:** 10.3389/fpls.2022.917910

**Published:** 2022-06-22

**Authors:** Yanbin Wen, Hongjiu Liu, Huanwen Meng, Lijun Qiao, Guoqing Zhang, Zhihui Cheng

**Affiliations:** ^1^College of Horticulture, Northwest A&F University, Xianyang, China; ^2^Development Center of Fruit Vegetable and Herbal Tea, Datong, China; ^3^Business School, Shanxi Datong University, Datong, China

**Keywords:** autopolyploid, colchicine, dwarfness, garlic, *in vitro*, oryzalin, whole-genome duplication

## Abstract

Garlic (*Allium sativum* L.) is a compelling horticultural crop with high culinary and therapeutic values. Commercial garlic varieties are male-sterile and propagated asexually from individual cloves or bulbils. Consequently, its main breeding strategy has been confined to the time-consuming and inefficient selection approach from the existing germplasm. Polyploidy, meanwhile, plays a prominent role in conferring plants various changes in morphological, physiological, and ecological properties. Artificial polyploidy induction has gained pivotal attention to generate new genotype for further crop improvement as a mutational breeding method. In our study, efficient and reliable *in vitro* induction protocols of autotetraploid garlic were established by applying different antimitotic agents based on high-frequency direct shoot organogenesis initiated from inflorescence explant. The explants were cultured on solid medium containing various concentrations of colchicine or oryzalin for different duration days. Afterward, the ploidy levels of regenerated plantlets with stable and distinguished characters were confirmed by flow cytometry and chromosome counting. The colchicine concentration at 0.2% (w/v) combined with culture duration for 20 days was most efficient (the autotetraploid induction rate was 21.8%) compared to the induction rate of 4.3% using oryzalin at 60 μmol L^–1^ for 20 days. No polymorphic bands were detected by simple sequence repeat analysis between tetraploid and diploid plantlets. The tetraploids exhibited a stable and remarkable dwarfness effect rarely reported in artificial polyploidization among wide range of phenotypic variations. There are both morphological and cytological changes including extremely reduced plant height, thickening and broadening of leaves, disappearance of pseudostem, density reduction, and augmented width of stomatal. Furthermore, the level of phytohormones, including, indole propionic acid, gibberellin, brassinolide, zeatin, dihydrozeatin, and methyl jasmonate, was significantly lower in tetraploids than those in diploid controls, except indole acetic acid and abscisic acid, which could partly explain the dwarfness in hormonal regulation aspect. Moreover, as the typical secondary metabolites of garlic, organosulfur compounds including allicin, diallyl disulfide, and diallyl trisulfide accumulated a higher content significantly in tetraploids. The obtained dwarf genotype of autotetraploid garlic could bring new perspectives for the artificial polyploids breeding and be implemented as a new germplasm to facilitate investigation into whole-genome doubling consequences.

## Introduction

Garlic (*Allium sativum* L.) is a diploid (2n = 2x = 16) bulb crop that has been cultivated for more than 5,000 years with high global demand and economic significance (Maaß and Klaas, 1995; Ricroch et al., 2005). It is widely consumed as condiment, green vegetable, and herbal medicine with various properties, such as antibacterial, antithrombotic, antioxidant, immunomodulatory, lipid-lowering, and antidiabetic actions (Ipek et al., 2005; Tsai et al., 2012; Liu et al., 2015; Ajami and Vazirijavid, 2019).

Although fertility restoration of garlic has been achieved, the commercial garlic varieties are still sterile due to pollen degeneration and reproduced vegetatively by planting individual cloves or bulbils (Pooler and Simon, 1994; Etoh and Simon, 2002; Simon and Jenderek, 2003; Shemesh Mayer et al., 2013; Shemesh-Mayer and Kamenetsky-Goldstein, 2021), and consequently, the classical hybridization in breeding strategy and genetic studies of this economically important crop has been strictly hindered for a long time (Shemesh-Mayer et al., 2015). Its germplasm resources for improvement are severely lacking.

Polyploidy, that is, the possession of three or more complete sets of chromosomes (Ramsey and Schemske, 1998), is one of the major moving forces in the evolutionary process of higher plants which promotes speciation, biodiversity, and adaptation to environmental alterations (Wood et al., 2009; Hegarty et al., 2013; Iannicelli et al., 2020). It was estimated that about 95% of ferns, 15% of gymnosperms, and 70% of angiosperms have experienced chromosomes doubling in their evolutionary history (Masterson, 1994; Wendel, 2000). Polyploidization has been demonstrated to introduce profound phenotypic alterations including morphological (Ramsey and Schemske, 2002; Ye et al., 2010; Catalano et al., 2021), physiological (Balal et al., 2017; Mattingly and Hovick, 2021), phytochemical (Pradhan et al., 2018; Zheng et al., 2021; Tavan et al., 2022), and molecular (Adams and Wendel, 2005; Chen and Ni, 2006; Yan et al., 2019; Zhang et al., 2022) characteristics. In recent years, polyploidization has become a powerful breeding strategy to enable the development of new and improved germplasm, even cultivars. According to the origination, polyploids normally can be classified as autopolyploids and allopolyploids (Trojak-Goluch et al., 2021).

There are two underlying mechanisms for the generation of polyploids: mitotic polyploidization by doubling the whole sets of chromosomes in meristematic cells developing to mixoploid or polyploidy organisms, and meiotic polyploidization which generates 2n gametes (Ramsey and Schemske, 1998; Sattler et al., 2016). Since the pioneering artificial polyploidy induction trial documented with colchicine (Blakeslee, 1922; Blakeslee and Avery, 1937), it has gained remarkable attention for agriculture, medicine, and horticulture utilization as safe and effective breeding strategy for the improvement of desired valuable properties, especially for the vegetatively propagated plants such as garlic (Dhooghe et al., 2011; Hailu, 2021). Colchicine (natural alkaloid) and oryzalin (synthetic herbicide) were most commonly used antimitotic agents (AMA) by inhibiting the metaphase in cell division cycle. The spindle of microtubules compiled of α- and β-tubulin dimers is crucial for controlling chromosome segregation and correct polar migration during cell division (Dewitte and Murray, 2003; Wu and Akhmanova, 2017). These two AMA disturb the metaphase by associating with the α- and β-tubulin dimers, thereby reducing the attachment of new dimers on the assembly side of the microtubule, without reducing degradation of the microtubule at the disassembly end. As a result, disassembly proceeds faster than assembly and microtubules are depolymerized (Dhooghe et al., 2011, and references therein). Consequently, inhibition of this chromosome separation results in cells with doubled chromosomes. There are extensive drawbacks of colchicine including high toxicity to humans, poor binding capacity to plant tubulins, and side effects such as sterility, abnormal growth, and chromosome losses (Morejohn et al., 1987; Luckett, 1989). Meanwhile, oryzalin, with a significantly reduced toxicity for humans, is also more affinitive for plant tubulin dimers than colchicine, which can therefore be used at lower concentrations (Dolezel et al., 1994; Ascough et al., 2008). Colchicine is generally applied in a concentration range of 1.25–2.5 mM, while other antimitotic agents as oryzalin, trifluralin, or APM have a final concentration of 1–50 μM (Dhooghe et al., 2011).

It has been demonstrated that there are multi-variant factors involved in APPI procedure turning the results unpredictable and nondeterministic (Dhooghe et al., 2011; Niazian and Nalousi, 2020). These factors mainly include AMA type (Koefoed Petersen et al., 2003; Zhang Y. S. et al., 2020), concentration and exposure duration of AMA (Allum et al., 2007; Ardabili et al., 2021), plant genotype (Stanys et al., 2006; Podwyszyńska et al., 2018; Ardabili et al., 2021), and application system as *in vivo* or *in vitro* (Rubuluza et al., 2007; Eng and Ho, 2019; Parsons et al., 2019). Consequently, the interaction among aforementioned parameters is ambiguously tangled making it impossible to declare that there is one optimal overall APPI protocol regarding specific species.

Due to the absence of adequate breeding method to introduce desirable variations, it has been a long time that new garlic varieties are selected only from existing living collections through natural or induced mutations (Shemesh-Mayer and Kamenetsky-Goldstein, 2021). Increasing ploidy by artificial polyploidy induction (APPI) is an efficient way to create superior plants to sterile plants such as garlic by improving the morphology, disease resistance, adaptability to environmental stress, and yield or quality (Balal et al., 2017; Zhou et al., 2020; Kim et al., 2021; Tavan et al., 2022). In spite of few reports concerning APPI for garlic germplasm innovation (Novák, 1983; Cheng et al., 2012), they mainly focused on induction protocols with single antimitotic chemical and basic ploidy determination instead of performance evaluation, especially physiological and phytochemical characteristics. Here, we established an effective induction system of autotetraploid garlic with multiple chemicals from inflorescence explants and conducted subsequent analysis of polyploidy effects on morphology, cytology, and physiology levels in a first reported dwarfness germplasms. This research lays important groundwork and provides a new perspective for the development of novel germplasm for garlic breeding efforts.

## Materials and Methods

### Plant Material

The widely grown garlic cultivar G064 was selected for artificial polyploidy induction. The healthy and uniform bulb cloves were cultivated in the garlic germplasm repository at the Horticultural Experimental Station (34°16′N, 108°4′E) of Northwest A&F University, Yangling, Shaanxi Province, China. Immature inflorescences with scape of 3 cm were collected as explant source when the ratio between scape length and pseudostem length was approximately 1 to 1.5 in late April to mid-May.

### *In vitro* Polyploidization of Garlic

Efficient and reproducible *in vitro* regeneration protocols are a prerequisite for efficient *in vitro* polyploidization systems (Niazian and Nalousi, 2020). We have established high-frequency direct shoot organogenesis protocols from garlic inflorescence in which the scape, sheathing bract, immature bulbils and flower or primordial residue on sterilized inflorescence were removed and the remainder was trimmed into dome shape explants aseptically (Wen et al., 2020). The explants were pre-cultured on shooting medium for 2 days to initiate cell division and facilitate synchronizing the cell cycle to maximize the effect of antimitotic agents (Touchell et al., 2020; Wen et al., 2020) and then transferred to shooting medium containing 0, 125, 250, 500, 1,000, or 2,000 mg L^–1^ colchicine or 0, 15, 30, 60, 120, or 240 μmol L^–1^ oryzalin for different durations (5, 10, 15, 20, 25, or 30 days). Dimethyl sulfoxide (DMSO) (0.02%) was added to medium to increase the penetration. Four explants were cultured in one bottle. The shooting medium was used as control. After induction treatment, the treated explants were retransferred to shooting medium and cultured for 20 days. The shooting medium was composed of B5-based solid medium supplemented with 6-BA 2 mg L^–1^ and NAA 0.1 mg L^–1^ adjusted to pH 7.0. The regenerated shoots were calculated and cultured on rooting medium for the initiation of roots and further growth of the intact regenerated plantlets. Rooting medium was MS medium 0.5 mg L^–1^ NAA adjusted to pH 7.0. All explants were cultured at 23 ± 2°C under cool-white fluorescent light by 16-h photoperiod with light intensity of 40 μmol m^–2^ s^–1^. The experiments were performed according to completely randomized design (CRD) with three replications per treatment (twenty explants per replicate). Data were analyzed using analysis of variance (ANOVA), and the difference between means was scored using LSD’s multiple range test by statistical package of SPSS (Version 17.0).

### Flow Cytometry Analysis

The determination of ploidy levels was conducted by flow cytometer (CytoFLEX, Beckman Coulter, Inc., United States). Appropriate nuclear isolation buffers are critical for preparation of suspensions of intact nuclei prior to analysis (Loureiro et al., 2006a). The Galbraith’s buffer (45 mM MgCl_2_, 20 mM MOPS, 30 mM sodium citrate, 0.1% (vol/vol) Triton X-100, adjusted to pH 7.0 with 1 M NaOH) (Galbraith et al., 1983) was selected after screening from six kinds of buffers (MgSO_4,_ Galbraith’s, LB01, Otto’s, GPB buffer, and Tris.MgCl_2_) accompanied by microscopic observations of nuclei suspensions. The intact nuclear suspensions were prepared from young single leaf in each sample according to Dolezel et al. (2007) and Pellicer et al. (2021). In brief, the nuclei extractions were released by chopping 100 mg leaf tissue quickly with a brand-new razor blade in a pre-cooled 60 × 15 mm petri dish containing 0.9 ml of Galbraith’s buffer followed by filtration through 30-μm nylon mesh to remove cell fragments and large tissue debris. Subsequently, 50 μl of 100 μg/ml propidium iodide (PI) and 50 μl of 100 μg/ml RNAse were added into the suspension to incubate in darkness for 10 min. PI was used to stain the nuclear DNA and RNase to eliminate the RNA and prevent the binding of PI to RNA (Loureiro et al., 2006b). Incubated samples were measured within 30 min by flow cytometer. CytoExpert 4.0 software was used for data analysis and outputting histogram of fluorescence intensities, which correspond to nuclear DNA contents. Genome sizes were measured on three non-consecutive days to ensure accuracy (Parsons et al., 2019). Four independent repetitions were performed on non-consecutive days to ensure accuracy, and at least 5,000 nuclei for each sample were analyzed.

### Chromosome Counting

For the determination of chromosome numbers in putative tetraploid plantlets by FCM, young healthy roots tips were immersed in 0.05 colchicine solution for 2 h at 0 – 4°C to accumulate metaphase cells and then fixed in Carnoy’s solution (ethanol: glacial acetic acid, 3: 1, v/v) for 24 h at 4°C. The fixed root tips were macerated by 1 mol/L HCL for 3 min in water bath at 60°C followed by rinsing with ice-cold water three times. The root tip cells were then excised and on a microscope slide following the squash method as described in a previous study and stained with a drop of Ziehl–Neelsen carbol fuchsin solution (Combination of 2.5 ml of melted phenol crystals, 5 ml of absolute alcohol, 0.5 g of basic fuchsin, and 50 ml of distilled water) (Lai and Lü, 2012). Cells were imaged using a compound microscope (Leica DM2000, Leica Microsystems, Heidelberg, Germany).

### Molecular Variance Analysis

The regenerated tetraploid plantlets and donor plants grown in the field of G064 were randomly selected to conduct the SSR analysis. Genomic DNA was extracted from 0.25 g freeze-dried young leaves following a modified cetyltrimethyl ammonium bromide (CTAB) protocol (Murray and Thompson, 1980). The quality and quantity of extracted DNAs were examined by electrophoresis in 1% agarose gel and measured using spectrophotometer (NanoDrop™ 2000/2000c, Thermo Fisher Scientific, United States), respectively. The DNA samples were diluted to 50 ng/ml in sterile distilled water. PCR amplifications were carried in 10 ml reactions, each containing 2 ml template DNA (50 ng/ml), 0.5 ml forward primers (5 mmol/l), 0.5 ml reverse primers (5 mmol/l), 2 ml ddH_2_O, and 5 ml 2 × Taq PCR Master mix (Tiangen Biotech Co., LTD., Beijing, China). The PCR program was as follows: 3 min at 95°C, 20 s denaturing at 94°C, 20 s annealing at 68°C, and 30 s elongation at 72°C, followed by a 2°C reduction in the annealing temperature per cycle for 6 cycles. Then, the annealing temperature was reduced in each cycle by 1°C for 8 cycles from 58°C; the annealing temperature was maintained at 50°C for the remaining 20 cycles, followed by a final step at 72°C for 5 min. The amplified PCR products were separated by vertical electrophoresis on 8% polyacrylamide gel in 1 × TBE buffer at a constant 180 V for 1 h, visualized with silver staining, and photographed with a digital camera. Twenty-nine developed SSR primers (Li et al., 2021) were selected. The tests were repeated twice to ensure reliability and repeatability.

### Morphological and Stomatal Characterization Analysis

Plantlets with different ploidy levels were sub-cultured for a further six months. Morphological studies of 10 diploid and tetraploid plantlets selected randomly were carried out. The well-developed functional leaves were used for measurement. The height, leaf length, leaf width, leaf thickness, leaf index, and root length were measured using an electronic digital caliper. Leaf pieces (5 mm^2^) were excised after 6 h of exposure to light within the growth chamber and processed for the cryo-scanning electron microscopy (cryo-SEM) (Hitachi FlexSEM1000, Japan) according to the protocol described by Kumar et al. (2012). The stomatal length, width, and density measurements were performed using ImageJ software^1^.

### Hormone Assessment

For hormonal assay, the levels of various hormones including indole-3-acetic acid (IAA), zeatin (ZT), dihydrozeatin (DHZT), abscisic acid (ABA), isopentenyladenosine (IPA), Brassinosteroid (BR), Methyl Jasmonate (MeJA), and gibberellins (GA_3_ and GA_4_) were evaluated using enzyme-linked immunosorbent assay (ELISA) technique as described in previous studies (Yang et al., 2001; Liu et al., 2018; Liu et al., 2020). The mouse monoclonal antigens and antibodies against ZR, IAA, ABA, GA_3_, and GA_4_ were provided by Phytohormones Research Institute (China Agriculture University, Beijing, China). The assays were run according to manufacturer’s instruction and in triplicate.

### Organosulfur Compound Evaluation

The content of organosulfur compounds including allicin, diallyl disulfides (DADS), and diallyl trisulfides (DATS) was determined using liquid chromatography (HPLC) method. The detailed procedure was described by Xu et al. (2012) with modifications. Basically, 0.2 g leaf samples were extracted with 1.6 ml ethanol by Motor-Driven Tissue Grinder (Tissuelyser-II, Jingxin Co., Ltd., Shanghai, China) and incubated at 95°C for 30 min followed by filtration through 0.22-μm membrane. The filtrate was isolated by YMC-Pack ODS-A C18 column (250 mm × 4.6 mm, 5 μm) with mobile phase consisting of acetonitrile-ultrapure water (70:30, v/v). The flow rate was set as 1.0 mL⋅min^–1^, and the UV wavelength was 240 nm.

## Results

### Induction of Tetraploid Garlic With Colchicine and Oryzalin

The results of analysis of variance (ANOVA) showed the significant main effect of colchicine concentration and applied exposure durations on viability, shoot regeneration ability, and tetraploid induction rate (Table 1). The total explant viability and shoot regeneration ability (regenerated shoots per explant, RSE) reduced along with the increasing levels of duration time and concentration of colchicine, which is not the case for tetraploid induction rate.

**TABLE 1:** Main effect of colchicine concentration and duration on viability of explants, shoot regeneration, and tetraploid induction rate from garlic inflorescence on solid medium.

Treatment	Viability (%)	Shoots/per explant	Tetraploid (%)
**Duration(d)**			
5	88.9 ± 2.9a	9.3 ± 1.2a	0.1 ± 0.5d
10	74.6 ± 4b	5.5 ± 0.9b	3.7 ± 2bc
15	60 ± 4.3c	4.2 ± 0.9c	6.2 ± 2.1bc
20	54.7 ± 3d	2.9 ± 0.7d	9.9 ± 2.7a
25	20.4 ± 3.1e	2.4 ± 1e	7.4 ± 2.4ab
30	7.3 ± 2.8f	0.9 ± 0.9f	2.8 ± 2.5c
**Conc. (mg L^–1^)**			
125	62.5 ± 5.5a	5.1 ± 1.8a	3.7 ± 2.3b
250	57.7 ± 5.5b	4.3 ± 1.6b	2.9 ± 2b
500	55.2 ± 5.8b	4.3 ± 1.7b	4.7 ± 2.2b
1000	44.7 ± 5.5c	3.9 ± 1.7bc	5.5 ± 2.2b
2000	34.7 ± 5.1d	3.5 ± 1.6c	8.2 ± 3a
**F-test**			
Duration	**	**	**
Conc.	**	**	*

Data with different letters in the same column indicate significant difference between means at the 5% probability level by LSD. *Indicates significant difference at 0.05 level (ANOVA and LSD’s multiple range test). **Indicates significant difference at 0.01 level (Two-directional ANOVA and LSD’s multiple range test).

The highest viability of 96.5% and RSE of 23.4 was observed from the application of 125 mg/L colchicine for 5 d but failed to induce tetraploid garlic (Table 2). The highest tetraploid induction rate was 21.8% achieved by highest concentration of 2,000 mg/L and 20 d duration. It is noticeable that no tetraploids were found with the concertation higher than 250 mg/L combined with the longest duration of 30 d, which was supported by the greater significance of duration (*) than that of concentration (**). Furthermore, there was significant interaction effect between concentration and duration on the viability and tetraploid induction rate except for RSE.

**TABLE 2:** Interaction effect of colchicine concentration and duration on viability of explants, shoot regeneration, and tetraploid induction rate from garlic inflorescence on solid medium.

Treatment		viability (%)	Shoots/per explant	Tetraploid (%)
Duration (d)	Concentration (mg L^–1^)			
0	0	100 ± 0	23.4 ± 1.4	0 ± 0
5	125	96.5 ± 1.7	11.3 ± 1.2	0 ± 0
5	250	95.2 ± 2.6	8.5 ± 0.9	0 ± 0
5	500	91.9 ± 2.7	9.5 ± 0.8	0 ± 0
5	1,000	85.3 ± 2.1	9.1 ± 0.6	0 ± 0
5	2,000	77.1 ± 2.2	7.9 ± 0.8	0.3 ± 0.7
10	125	91.7 ± 2.8	6.5 ± 1	0 ± 0
10	250	81.7 ± 1.7	5.3 ± 0.8	0 ± 0
10	500	86.8 ± 2.8	5.5 ± 0.5	3.3 ± 1.7
10	1,000	67.2 ± 3.1	5.4 ± 0.8	5.9 ± 1.4
10	2,000	45.5 ± 3.1	4.8 ± 0.8	9.2 ± 1
15	125	74.1 ± 2	5.1 ± 0.8	2.3 ± 0.4
15	250	69.7 ± 2.6	4.6 ± 0.6	3.1 ± 1
15	500	72.8 ± 2	4 ± 0.8	4.2 ± 1
15	1,000	53.4 ± 2.2	3.9 ± 0.9	10.2 ± 1
15	2,000	29.9 ± 3.4	3.3 ± 0.9	11.3 ± 2
20	125	63.3 ± 2.4	3.5 ± 0.3	2.6 ± 0.7
20	250	62.2 ± 2.1	2.5 ± 0.6	4.7 ± 1.4
20	500	55.5 ± 2.5	3 ± 0.5	10.6 ± 1.8
20	1,000	47.8 ± 2.5	2.8 ± 0.7	10 ± 0.6
20	2,000	44.6 ± 1.2	3 ± 0.5	21.8 ± 2.1
25	125	31.2 ± 1.8	2.7 ± 0.6	7.5 ± 1.4
25	250	28.6 ± 1.2	2.7 ± 0.7	5.7 ± 2.4
25	500	18.4 ± 1.4	3.2 ± 1	10.4 ± 1.3
25	1,000	13.1 ± 3.5	1.7 ± 1.2	6.7 ± 2.4
25	2,000	11 ± 1.7	1.7 ± 0.9	6.7 ± 3.4
30	125	18.3 ± 2.4	1.6 ± 0.7	10 ± 3.2
30	250	10.6 ± 2.4	1.8 ± 0.5	4.2 ± 2.7
30	500	6 ± 2.3	0.8 ± 0.9	0 ± 0
30	1,000	1.4 ± 1.6	0.3 ± 0.8	0 ± 0
30	2,000	0 ± 0	0 ± 0	0 ± 0
Duration × Conc.		**	NS	**

Data with different letters in the same column indicate significant difference between means at the 5% probability level by LSD. **Indicates significant difference at 0.01 level (Two-directional ANOVA and LSD’s multiple range test).

As for oryzalin treatment, the results also showed significant main effect from duration on viability, RSE, and tetraploid induction rate, while concentration did not present significant main effect on tetraploid induction rate (Table 3). Increasing concentrations and exposure durations also led to the significant reduction in viability and RSE. The most efficient way for chromosome doubling was found to be exposed to 60 μmol L^–1^ oryzalin for 20 days with induction rate of 4.3% (Table 4). Unlike colchicine, the concentration and duration of oryzalin had no significant interaction effect on tetraploid induction rate but for viability and RSE.

**TABLE 3:** Main effect of oryzalin concentration and duration on viability of explants, shoot regeneration, and tetraploid induction rate from garlic inflorescence on solid medium.

Treatment	Viability (%)	Shoots/per explant	Tetraploid (%)
**Duration (d)**			
5	97a	18.4a	0.2d
10	91.7ab	15.8b	1.5c
15	87.7c	15.1b	2.1bc
20	78.3d	13.9c	3.2a
25	65.5e	9.5d	2.7ab
30	46.3f	8.2e	1.8bc
**Conc. (μmol L^–1^)**			
15	85.6a	15.6a	1.7a
30	83.1a	14.9a	1.8a
60	76.5b	14.6a	2.2a
120	74.2bc	11.7b	2.1a
240	69.4c	10.5c	1.7a
**F-test**			
Duration	**	**	**
Conc.	**	**	NS

Data with different letters in the same column indicate significant difference between means at the 5% probability level by LSD. **Indicates significant difference at 0.01 level (Two-directional ANOVA and LSD’s multiple range test).

**TABLE 4:** Interaction effect of oryzalin concentration and duration on viability of explants, shoot regeneration, and tetraploid induction rate from garlic inflorescence on solid medium.

Duration	Concentration	Viability (%)	Average Shoots/per explant	Tetraploid (%)
0	0	100 ± 0	23.4 ± 1.4	0 ± 0
5	15	100 ± 0	19.5 ± 1.1	0.0 ± 0
5	30	98.3 ± 1.7	18.8 ± 0.5	0.0 ± 0
5	60	96.7 ± 1.7	18.4 ± 1.0	0.3 ± 0.3
5	120	95.0 ± 2.9	18.3 ± 0.6	0.2 ± 0.1
5	240	95.0 ± 2.9	17.1 ± 0.6	0.5 ± 0.3
10	15	96.7 ± 1.7	18.2 ± 0.6	0.8 ± 0.5
10	30	96.7 ± 1.7	15.9 ± 0.2	0.9 ± 0.5
10	60	91.7 ± 4.4	18.4 ± 1.7	1.5 ± 0.2
10	120	88.3 ± 4.4	13.5 ± 0.3	2.0 ± 0.8
10	240	85.0 ± 2.9	13.2 ± 1.2	2.4 ± 0
15	15	93.3 ± 4.4	15.5 ± 0.3	1.4 ± 0.7
15	30	91.7 ± 4.4	16.7 ± 1.4	1.6 ± 0.5
15	60	86.7 ± 1.7	17.4 ± 1.3	2.0 ± 0.4
15	120	86.7 ± 3.3	13.2 ± 1.0	2.8 ± 0.6
15	240	80.0 ± 2.9	12.6 ± 1.0	2.5 ± 0.7
20	15	78.3 ± 1.7	15.7 ± 0.7	2.8 ± 0.7
20	30	80.0 ± 0	16.1 ± 0.7	2.6 ± 0.3
20	60	81.7 ± 4.4	16.1 ± 0.5	4.3 ± 0.4
20	120	78.3 ± 3.3	11.1 ± 1.1	3.4 ± 1.1
20	240	73.3 ± 1.7	10.5 ± 1.1	2.8 ± 1.1
25	15	73.3 ± 1.7	13.1 ± 0.7	2.2 ± 0.3
25	30	73.3 ± 1.7	11.9 ± 0.8	3.4 ± 1.4
25	60	75.0 ± 5.8	9.1 ± 1.1	3.2 ± 0.9
25	120	68.3 ± 4.4	7.4 ± 0.4	2.3 ± 0.7
25	240	61.7 ± 3.3	5.9 ± 0.6	2.3 ± 0.9
30	15	71.7 ± 6.0	11.8 ± 1.3	2.7 ± 0.7
30	30	58.3 ± 4.4	10.2 ± 0.7	2.3 ± 1.2
30	60	51.7 ± 1.7	8.5 ± 1.1	2.1 ± 1.3
30	120	28.3 ± 3.3	6.7 ± 0.6	1.9 ± 0.9
30	240	21.7 ± 3.3	4.0 ± 0.8	0.0 ± 0
Duration × Conc.		*	*	NS

Data with different letters in the same column indicate significant difference between means at the 5% probability level by LSD. *Indicates significant difference at 0.05 level (ANOVA and LSD’s multiple range test).

### Ploidy Assessment

#### FCM Analysis

**FIGURE 1:**
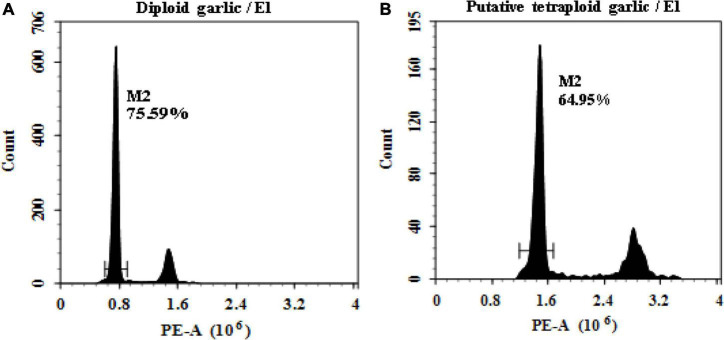
Linea histograms of the relative fluorescence intensity of garlic with FCM. **(A)** Diploid (2n = 2x = 16). **(B)** Putative tetraploid (2n = 2x = 32).

The ploidy level of regenerated plantlets was evaluated by flow cytometry. It proved that flow cytometry was a fast and reliable screening method of ploidy level. The results indicated that linear histograms of relative nuclear DNA content of diploid control showed distinctive G0/G1 peaks at channel 0.8, whereas the induced mutant plantlets showed the same peak at channel 1.6 which was defined as putative tetraploid (Figure 1).

#### Chromosome Counting

**FIGURE 2:**
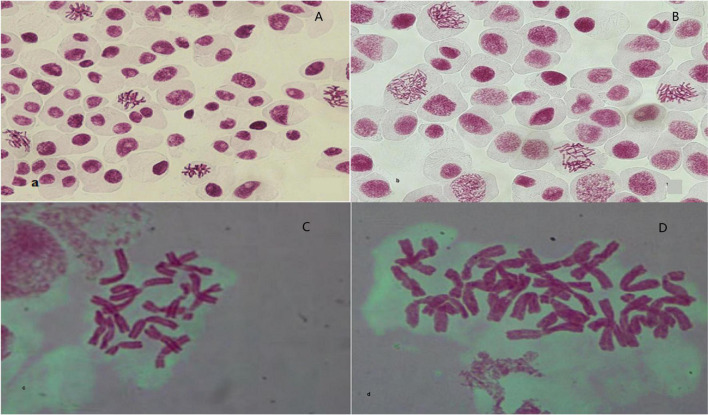
Observation and validation of root tip chromosomes in regenerated plantlets. **(A,C)** Diploid garlic (2n = 2x = 16). **(B,D)** Tetraploid garlic (2n = 2x = 32).

After confirming the increase in genome size and separating tetraploid plants from diploids using flow cytometry, the microscopic chromosomal counting method was used. In this study, the chromosome number obtained for diploid plants was 2n = 2x = 16 and for tetraploid plants was 2n = 4x = 32 (Figure 2).

#### SSR Analysis

**FIGURE 3:**
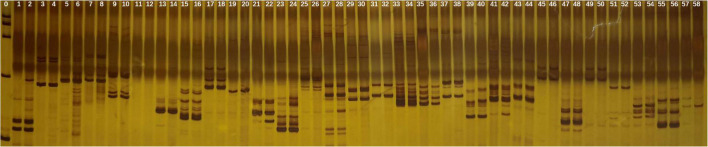
SSR banding pattern in the tetraploid and diploid garlic plantlets by 29 primers. Lane 0: marker, odd lane: diploid garlic, even lane: tetraploid garlic.

Of the 29 primers tested in SSR analysis, 28 markers presented clear, strong, and repeatable bands. These markers produced 65 scorable bands. The bands varied from one to five for each primer. No polymorphic bands were observed which indicated that neither gain nor loss of DNA sequence occurred after polyploidization of garlic in comparison with diploid (Figure 3).

### Morphological Comparison

**FIGURE 4:**
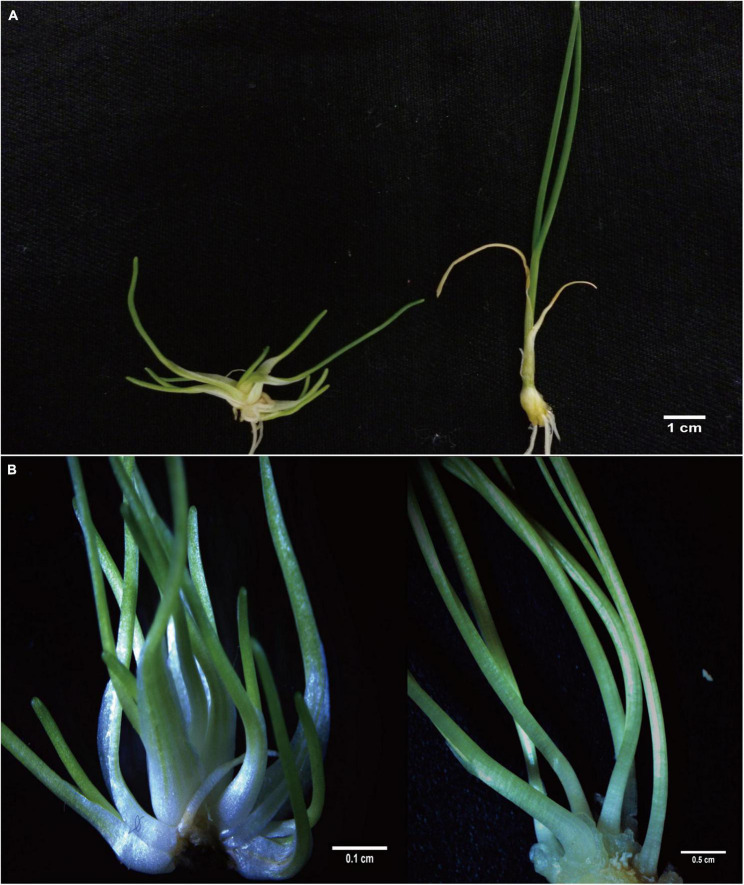
Comparison of plant architecture and growth characteristics of diploid and tetraploid in different regeneration stages. **(A)** Developed tetraploid (left) and diploid (right) plantlet. **(B)** Newly regenerated tetraploid (left) and diploid (right) shoots.

We found that all the autotetraploids induced from different AMA exhibited stable and similar uniform dwarf characteristics without pseudostem but densely packed leaves, and remarkably slow growth after successive culture for more than 2 years (Figure 4). The tetraploid showed significantly shorter and wider leaf by 1.7 and 1.5 times, respectively, which resulted in a significant decrease in leaf index compared to diploid (Table 5). The leaf thickness also increased by 3.5 times on average. The shorter root length was observed in tetraploids with developmental sluggish.

**TABLE 5:** Morphological characteristics of diploid and tetraploid garlic.

Morphological characteristics	Leaf length (mm)	Leaf width (mm)	Leaf index	Leaf thickness (mm)	Root length (mm)
Diploid	50.9 ± 6.2**	3.4 ± 0.3	14.9 ± 0.6**	0.4 ± 0.0	112.0 ± 2.3**
Tetraploid	18.2 ± 1.2	8.4 ± 0.4**	2.2 ± 0.1	1.8 ± 0.1**	71.4 ± 3.4

Data are presented as mean ± SE. Data with **Indicate significant difference at 0.01 level by one-directional ANOVA and Student’s t-test.

### Stomatal Variation

**FIGURE 5:**
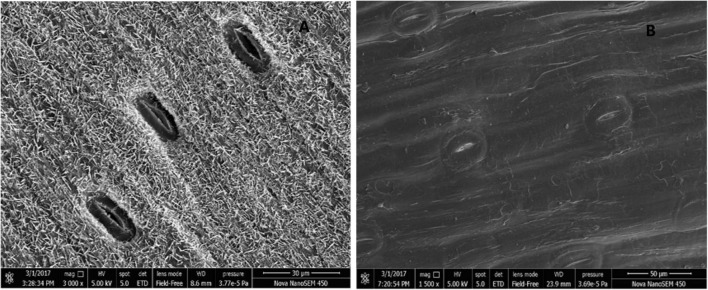
Cryo-scanning electron micrographs of stomata. **(A)** Diploid garlic. **(B)** Tetraploid garlic.

Tetraploid induction led to significant changes in stomatal traits. The absence of waxy layer on leaf surface was observed (Figure 5). The width and area of abaxial stomata apparatus increased by 43 and 53% in tetraploids, while the density significantly reduced by 2 times. No difference was observed in the stomata length (Table 6).

**TABLE 6:** Characteristics of stomatal apparatus in diploid and tetraploid garlic plantlets.

Ploidy level	Length (μm)	Width (μm)	Area (μm^2^)	Density (mm^2^)
Diploid	35.2 ± 3.2	12.2 ± 1.7	324.7 ± 50.2	1.30E-04**
Tetraploid	35.3 ± 4.2	17.4 ± 3.0**	497.1 ± 109.2**	4.55E-05

Data are presented as mean ± SE. **Indicates significant difference at 0.01 level by one-directional ANOVA and Student’s t-test.

### Physiological and Sulfur-Containing Compound Evaluation

**FIGURE 6:**
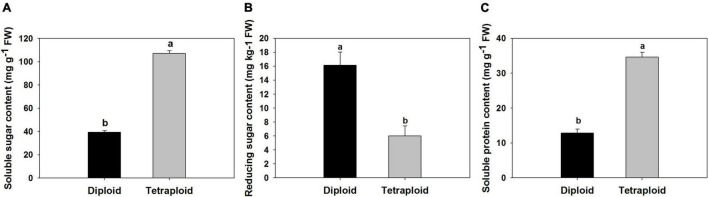
Comparison of physiological compounds. **(A)** Soluble sugar. **(B)** Reducing sugar. **(C)** Soluble protein.

**FIGURE 7:**
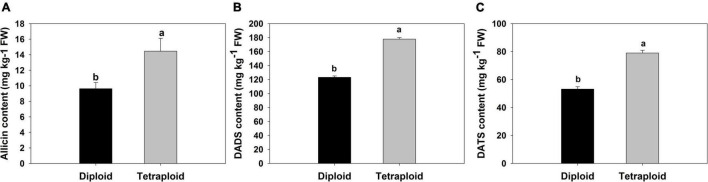
Comparison of sulfur-containing compounds. **(A)** Allicin, **(B)** DADS. **(C)** DATS.

A comparison of physiological parameters in diploid and tetraploid plants showed significant differences. The content of total soluble sugar and protein increased by 172 and 166% in tetraploids, On the contrary, the amount of reduced sugar was found 1.7 times higher in diploid plants than in tetraploid (Figure 6). Allicin, diallyl disulfide (DADS), and diallyl trisulfide (DATS) are the main active metabolites in garlic, and HPLC analysis showed that allicin, DADS, and DATS were 50%, 44.3%, and 48.6% higher than those of diploids, respectively (Figure 7).

### Endogenous Hormone Analysis

**FIGURE 8:**
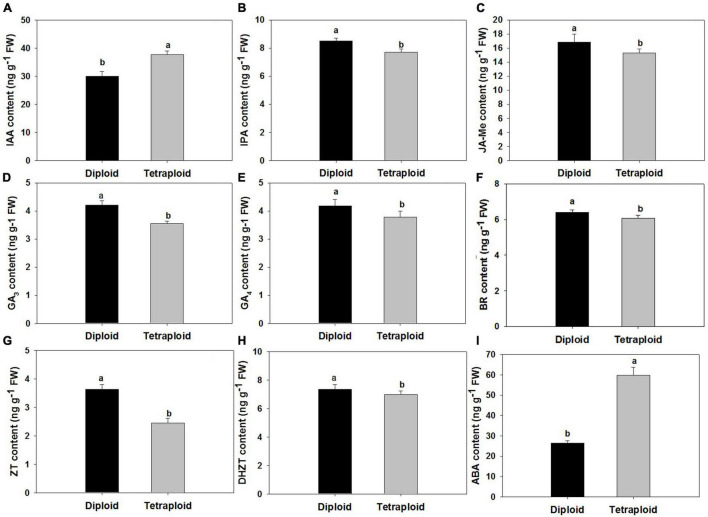
Comparison of endogenous hormone levels. **(A)** Indole-3-acetic acid (IAA). **(B)** Isopentenyladenosine (IPA). **(C)** Methyl Jasmonate (MeJA). **(D)** Gibberellins (GA_3_) **(E)** Gibberellins (GA_4_). **(F)** Brassinosteroid (BR). **(G)** Zeatin (ZT). **(H)** Dihydrozeatin (DHZT). **(I)** Abscisic acid (ABA).

Compared with diploid, the contents of nine endogenous hormones in the autotetraploid garlic as indole acetic acid (IAA), indole propionic acid (IPA), gibberellin (GA_3_ and GA_4_), abscisic acid (ABA), brassinolide (BR), zein (ZT), dihydrozeatin (DHZT), and methyl jasmonate (MeJA) changed significantly. The elevated contents of IAA and ABA were detected in tetraploids which increased significantly, while the contents of other hormones were slightly lower than those in diploids (Figure 8).

## Discussion

### Chromosome Doubling

Autopolyploidization, formed by within-species whole-genome duplication, was widely considered to be a massive mutation force in plant evolution and powerful tool to provide a broad germplasm base in breeding program (Sattler et al., 2016; Bohutínská et al., 2021). Although the artificial induction of polyploidy has been used to introduce comprehensive alterations in morphological, histological, physiological, agronomic, and genomic levels in plenty of plant species, the efficiency of specific protocols and consequences are still ambiguous (Sanwal et al., 2010; Zhang et al., 2010; Rambani et al., 2014; Ahmadi and Ebrahimzadeh, 2020; Baghyalakshmi et al., 2020; Fox et al., 2020).

Among the polyploidy induction methods, *in vitro* techniques can increase the efficiency of polyploidy induction and reduce mixoploid formation by minimizing the complex influence from internal or external factors and guarantee the multiplication of the mutant throughout the year (Touchell et al., 2020; Bhusare et al., 2021). The uniformity of environmental factors such as temperature and light can simultaneously cause meristem cell division, decrease mixoploid progeny, and increase complete tetraploid progeny (Niazian and Nalousi, 2020).

Stable and efficient regeneration system is a prerequisite for polyploidy induction or genetic manipulation (Wang et al., 2011). Nowadays, the most commonly used explants for *in vitro* polyploidy induction are shoot tips, axillary buds, petiole or leaf explants, nodal segments, roots, and callus (Trojak-Goluch et al., 2021). The main problems in mutation breeding of vegetatively propagated plants *in vitro* are the formation of chimeras and the somatic elimination (diplontic selection) of mutated sectors after mutagenic treatment (Suprasanna et al., 2015). Consequently, it is suggested that the best method for *in vitro* polyploidization is through direct adventitious shoot regeneration without pre-existence of shoot-bud or meristem tissue, because young meristematic portions are more flexible to polyploidy induction, since they provide better permeability to the antimitotic chemicals (Eng and Ho, 2019; Touchell et al., 2020). Satisfactory results of polyploidy induction are also obtained as a result of antimitotic treatment of seedlings containing intensely dividing meristematic tissues for many industrial species (Trojak-Goluch et al., 2021). To establish the polyploidization system, we explored a high-frequency direct shoot organogenesis from garlic inflorescence as explant with active meristematic status, which is more amenable to ploidy alterations and in regeneration of autopolyploid shoots (Wen et al., 2020; Gantait and Mukherjee, 2021).

Utilization of suitable antimitotic chemicals is critical in polyploidy induction. Among different antimitotic agents, colchicine has been the most commonly used chemical as the criteria (Dhooghe et al., 2011). However, it also causes side effects such as sterility, abnormal growth, chromosome losses or rearrangements, and gene mutation (Luckett, 1989). Moreover, due to its high affinity to microtubules of animal cells, colchicine is highly toxic to mammals and impacts negatively on the environment (Morejohn et al., 1987). Colchicine binds poorly to plant tubulins; thus, it is usually used in relatively high concentrations. For these multiple drawbacks, mitosis-inhibiting herbicides with more affinity for plant tubulin dimer have gained attraction as its alternatives (Wan et al., 1991; Häntzschel and Weber, 2010). They have outperformed colchicine for the higher efficiency of polyploidy induction in many plant species including fruit (van Duren et al., 1996; Bartish et al., 1998), vegetable (Viehmannová et al., 2009), ornamentals (Van Laere et al., 2006), agricultural crops, and forage (Quesenberry et al., 2010). However, our study demonstrated that colchicine is still the optimal mutation agent with tetraploid induction rate of 21.8% compared to 4.3% for oryzalin.

The concentration and exposure time of specific antimitotic compounds are most crucial factors. Too low doses may be ineffective, while excessively high concentrations are toxic and usually cause reduced viability even lethal. Furthermore, high concentrations and exposure times can result in higher ploidy levels than desired (Allum et al., 2007). Up to now, consistent investigation has established the agreement that in case of successful induction treatment, usually lower concentrations were accompanied by longer exposure duration and vice versa. One cannot proclaim that one antimitotic agent is the most successful (Dhooghe et al., 2011) even for certain species, because it is also significantly affected by the genotype and explant type of the donor plant (Trojak-Goluch et al., 2021). The evaluation of main and interaction effect between these two factors has yet been conducted in related research before. We demonstrated that the concentration of oryzalin had no main effect on tetraploid induction rate in agreement with its high affinity; meanwhile, the interaction effect was not observed neither. It means the duration scale setting should be paid more concise considerations.

### Morphological and Hormonal Variations

Genome doubling event is a single macromutation with many phenotypic consequences (Doyle and Coate, 2020). Autopolyploids tend to be superior to their diploid parents with respect to morphological changes, genetic adaptability, and tolerance to environmental stresses (Leitch and Leitch, 2008), among which, the giga effect as bigger organs was preeminent. However, polyploids do not always exhibit higher quality and/or enlargement (Tsai et al., 2021). It illustrates that stochastic changes in phenotype initiated by ploidy are species-dependent (Trojak-Goluch et al., 2021). The autotetraploid garlic obtained from our study is accompanied by thicker, wider, but much shorter leaves and developmentally delayed roots reported previously (Dixit and Chaudhary, 2014; Tavan et al., 2015; Ayu et al., 2019), along with difficulty acclimating to a greenhouse environment (Roy et al., 2001; Trojak-Goluch and Skomra, 2013).

Dwarfing is the most noticeable phenotype displayed in tetraploid garlic compared to the diploid counterpart. The extreme dwarfness after tetraploidization was also reported in apple (Ma et al., 2016), cabbage (Ari et al., 2015), Chinese jujube (Wang et al., 2019), Escallonia genus (Denaeghel et al., 2018), and Z. *zamiifolia* (Seneviratne et al., 2020) following genome doubling.

Polyploidization can alter plant morphology, phenology, and physiology within only one or a few generations (te Beest et al., 2012). After more than two consecutive years of subculture, we found that newly regenerated autotetraploid garlic plantlets were still lack of pseudostem and significantly shorter than the diploid. We thus excluded the possibility that colchicine or oryzalin toxicity caused dwarfism of autotetraploid garlic.

The dwarfness of tetraploid garlic could attribute to its slower growth (Rao et al., 2019; Yan et al., 2022). Following polyploidization, individuals may experience “genomic shock” (McClintock, 1984) conferring disruption of the balance between nuclear and cytoplasmic components which inhibits the completion of mitosis and meiosis (Cavalier-Smith, 1978; Otto and Whitton, 2000; Otto, 2007; Manzoor et al., 2019). It was suggested that increasement of cell volume after polyploidization could strongly reduce cell division rate and slow down the activity of metabolism, consequently resulting in low growth rates (Cavalier-Smith, 1978; Tsukaya, 2013; Corneillie et al., 2019; Sabooni et al., 2022).

Recent reports have demonstrated that dwarfism and organ development in polyploidy were regulated by the complex interaction of various phytohormones (Kondorosi et al., 2000; Wang Y. et al., 2018). It has been reported that mutants with defects in plant hormone biosynthesis or signaling could result in dwarfism (Durbak et al., 2012; Chen et al., 2016; Wang B. et al., 2018). Most dwarfism phenotypes in plants are associated with genes involved in the biosynthesis or signaling pathways of GA, BR, and IAA (Nemhauser et al., 2006; Wang and Li, 2008; Ma et al., 2016; Wang B. et al., 2018).

The deficiency of active GAs, brassinosteroids (BRs), was detected in the dwarfism phenotype for tetraploid rice, apple, and Arabidopsis which applied with our results (Spielmeyer et al., 2002; Yin et al., 2002). Studies have convincingly demonstrated that GA deficiency specifically causes the decrease in plant height. The dwarf and semidwarf rice resulted from a deficiency in active GAs (Spielmeyer et al., 2002; Sakamoto et al., 2004). The impaired GA biosynthesis was found in the dwarf banana phenotypes (Chen et al., 2016; Shao et al., 2020). Decreased accumulation of GAs can suppress additional cell divisions and decrease the size of the division zone thereby inhibiting leaf growth and contributing to the semidwarf phenotype in maize (Nelissen et al., 2012; Zhang J. et al., 2020). The mutants defective in BR synthesizing genes also reduce plant height in rice, sorghum, and barley (Fujioka et al., 1998; Dockter and Hansson, 2015; Hirano et al., 2017). This could be attributed to the inhibition of genes, transcription factors, or enzymes related to BR synthesis and signal transduction pathways which affect the cell elongation or expansion (Nakamura et al., 2006; Zhao et al., 2022).

The IAA and ABA contents were significantly higher in tetraploid dwarf garlic. The significantly higher IAA was found in the dwarf bananas which likely regulated GA biosynthesis by negative feedback (Deng et al., 2021). IAA was also found to induce the overexpression of OsIAA1 gene (member of Aux/IAA and auxin response factor), leading to shorter plant and loose architecture distinctively in rice (Song et al., 2009). ABA accumulation by higher transcript level of the ABA pathway genes exhibited the dwarf yellowing phenotype in pear which is also consistent with our findings (Pang et al., 2019). It was illustrated that high concentrations of ABA not only inhibit cell division in the apical meristems but also repress the elongation of roots (Bai et al., 2009; Takatsuka and Umeda, 2014). Expression of key genes related to ABA was significantly upregulated in cabbage dwarf mutant (Xing et al., 2020). Controversially, the higher IAA but lower ABA contents in dwarf autotetraploid Chinese Cabbage was found (Wang Y. et al., 2018). In addition to the abovementioned hormones, the low content of MeJA also caused dwarf traits in rice (Gan et al., 2015).

Our results of various endogenous hormones in diploid and tetraploid garlic provided partial explanation of morphological alterations (Maru et al., 2021). It should be noted that the factors affecting plant height are complex and diverse. Noticeably, Xing et al. (2020) implied that the change in phytohormones is due to but not the cause of the dwarf trait in polyploidy cabbage. Further studies of transcriptome analysis are worth pursuing to facilitate a better understanding of dwarfism mechanism in tetraploid garlic.

### Functional Metabolites

It is widely acknowledged that multiplication of genome bestows conspicuous enhancement of nutritional and secondary metabolites yield, which has contributed to a significant commercial value for industrial and medicinal importance (Gantait and Mukherjee, 2021). The significantly higher bioactive constituents of total flavonoid and gastrodin in all parts of tetraploid *Anoectochilus formosanus Hayata* were evaluated compared with the diploid (Chung et al., 2017). It also demonstrated a dramatic change in secondary metabolism (terpene composition) related to an increase in the ploidy level in Eucalyptus germplasms (da Silva et al., 2021). Nevertheless, in *Solanum bulbocastanum*, the lower contents of phenylpropanoids, tryptophan, and tyrosine were found in tetraploids than in diploids (Caruso et al., 2011). This indicates that genome doubling does not increase the accumulation of high-value bioactive compounds all the time. In this study, the dwarfing tetraploid plantlets showed higher levels of allicin, DADS, and DATS than diploid counterpart, suggesting the potentiality as a breeding method in garlic for abundant production of pharmaceuticals.

## Conclusion

In this study, a successful *in vitro* polyploidization protocol was established with colchicine and oryzalin in garlic. The colchicine led to the highest tetraploid induction rate of 21.8% with the application of 200 mg/L for 20 days. The unexpected dwarfing tetraploids were characterized for their morphological traits and phytochemical variations. This is the first report suggesting that chromosome doubling could impart garlic with dwarfism. Our study provides a valuable germplasm resource to broaden the elucidation of polyploidization consequences, as well as the genetic studies and possible major breakthrough for future improvement of Allium species.

## Data Availability Statement

The raw data supporting the conclusions of this article will be made available by the authors, without undue reservation.

## Author Contributions

YW conceived the idea, designed and performed the experiment, and prepared the draft manuscript. HL participated in the design of the study, carried out experiments, and reviewed the manuscript. HM contributed to analysis of the data. LQ conducted molecular variance analysis. GZ contributed to data analysis, revising, and funding acquisition for publication. This work was conducted under the supervision of ZC who provided significant intelligence to the manuscript revision. All authors contributed to the article and approved the submitted version.

## Conflict of Interest

The authors declare that the research was conducted in the absence of any commercial or financial relationships that could be construed as a potential conflict of interest.

## Publisher’s Note

All claims expressed in this article are solely those of the authors and do not necessarily represent those of their affiliated organizations, or those of the publisher, the editors and the reviewers. Any product that may be evaluated in this article, or claim that may be made by its manufacturer, is not guaranteed or endorsed by the publisher.

## Footnotes

1
http://rsb.info.nih.gov/ij/
